# Taxonome: a software package for linking biological species data

**DOI:** 10.1002/ece3.529

**Published:** 2013-04-01

**Authors:** Thomas A Kluyver, Colin P Osborne

**Affiliations:** Department of Animal and Plant Sciences, University of SheffieldSheffield, United Kingdom

**Keywords:** Binomials, fuzzy matching, name matching, synonyms

## Abstract

Online databases of biological information offer tremendous potential for evolutionary and ecological discoveries, especially if data are combined in novel ways. However, the different names and varied spellings used for many species present major barriers to linking data. *Taxonome* is a software tool designed to solve this problem by quickly and reproducibly matching biological names to a given reference set. It is available both as a graphical user interface (GUI) for simple interactive use, and as a library for more advanced functionality with programs written in Python. *Taxonome* also includes functions to standardize distribution information to a well-defined set of regions, such as the TDWG World Geographical Scheme for Recording Plant Distributions. In combination, these tools will help biologists to rapidly synthesize disparate datasets, and to investigate large-scale patterns in species traits.

## Introduction

People have studied living organisms for centuries, recording much of the information at the level of species. In the 21st century, these data are increasingly placed online, whether in well-curated databases (e.g. Royal Botanic Gardens, Kew 2008; Missouri Botanical Garden 2012) or forgotten spreadsheets. Many interesting analyses hinge upon combining data from different sources using the scientific names of species, and this approach offers the potential for major advances in understanding (Sidlauskas et al. 2010). However, 250 years of taxonomic revisions and spelling mistakes present a major obstacle to linking datasets. For small numbers of species, the links can found manually, but the process is frustrating, time-consuming, and the results cannot be readily reproduced. An automated matching process is therefore highly desirable, and is essential for large datasets.

Most species are identified by a Linnaean binomial name (Linnaeus 1753; Patterson et al. 2010), but these have a number of undesirable features for automatic matching. Some authors have proposed an entirely new system of numeric identifiers for taxa (Page 2009), but so far no such scheme is in widespread use. Even if numeric identifiers were to be adopted, tools would still be needed to apply them to existing data.

The challenges for computer systems handling taxonomic names are:

- Synonymy: Many taxa have been given several names, either because authors were unaware of earlier descriptions, or because of taxonomic revisions. For some groups, reasonably comprehensive synonymies are available (e.g. grass species names compiled by Clayton et al. (2002)).
- Homonymy: One name may have been applied to more than one species, for instance the name *Glycyrrhiza glandulifera* has been used for the species now called *Glycyrrhiza glabra* and *Glycyrrhiza uralensis*. More rigorous sources give the name with an author citation, such as “*Glycyrrhiza glandulifera* Ledeb”, which can be used to find the correct match.
- Spelling differences: People may make mistakes transcribing a name, but there are also long standing variations in spelling, such as *Triticum baeoticum* or *boeoticum*. The requirement that a specific epithet agree with the gender of its genus leads to confusions such as *Viscum alba* (instead of *Viscum album*). These variations are often not listed as synonyms. Spelling differences in author citations are a more common problem; in botany there is a standard list of author abbreviations (Brummitt and Powell 1992; International Plant Names Index 2008), but many sources do not follow this convention.
- Data formats: Most biodiversity datasets are not available in standard formats such as Darwin Core (Darwin Core Task Group 2009). Data are often stored in comma-separated value (CSV) files, but this simple format encompasses many possibilities – such as combining the binomial name and author citation into one field, or separating them.

## Methods

Taxonome has been developed to handle and match scientific names automatically, following standard taxonomic rules. It uses fuzzy matching to account for spelling variations or mistakes. While initial development focused on plant nomenclature (McNeill et al. 2006; Miller et al. 2011), it is also flexible enough to deal with zoological names (International Commission on Zoological Nomenclature 1999), although the two systems use slightly different formats.

Taxonome treats a taxon as having one accepted name (as described by the chosen data source), and a number of synonyms. Each taxon may also have other associated information, such as its distribution and data about biological traits. A group of taxa from one source are stored in a data structure (a TaxonSet) which indexes all the names, so that a taxon can be quickly found given a binomial name.

Where separate data sources have information on the same taxa, these are represented as two separate collections, and one may be matched against the other. Matching preserves the information attached to each taxon, but reassigns its name to the accepted name from the dataset against which it is matched. The matching process can also produce CSV files recording the matches made and the different steps taken. Several collections of taxa with matched names may then be combined into one set.

To match a name, a number of possibilities are tried, most of them user-configurable:

- An exact match, including the authority, is always preferred.
- If a name matches but does not have a matching authority, this can be used unless the user has disabled such matches. However, if the authorities specifically indicate that the names refer to different taxa, the match is rejected (see below).
- Taxa below species level which do not have an exact match can be matched to the parent species. This can be done for all subspecies, only for nominal subspecies (e.g. *Zea mays* subsp. *mays*), or disabled.
- Where possible, fuzzy matching is used to account for spelling variations and errors in the data (see below).

In the case of homonyms, more than one match may be found. If one of the matches is an accepted name, Taxonome can accept it as the most likely option. This is done by default when the name being matched does not have author information. Otherwise, the matching process can be set to let the user decide in such cases. The user can pick from the available matches, enter a replacement name, or reject all the options.

Taxonome employs fuzzy string matching to account for differences in spelling. For binomial names, an approach based on q-grams is used (Gravano et al. 2001). Each name is broken into overlapping chunks of three letters, including two padding characters at the beginning. The standard q-gram algorithm also includes padding characters at the end, but Taxonome omits these to give less weight to the ending, where the spelling most often differs. The proportion of these chunks which another name has in common gives a similarity score. To speed up lookups, the first three characters of the name must match exactly. For example, if no exact match is found for *Mucuna holtoni*, it is broken down to ‘∧∧M’, ‘∧Mu’, ‘Muc’, ‘ucu’, etc. The set of q-grams is then compared with those for each name beginning with ‘Muc’, finding a 93% overlap with the q-grams for *Mucuna holtonii* (with a double i). By contrast, *Mucuna restonii* only shares 60% with *Mucuna holtonii*, below the default acceptance threshold of 70%. This threshold can be altered by the user.

For author citations, which are typically very short strings, a more bespoke approach is used. Taxonome identifies components such as initials, surnames, and dates. This is particularly important when a name is qualified with a phrase like ‘non Vahl’, which means that it is not the name defined by Vahl. A simple string similarity test might erroneously match with ‘Vahl’, but Taxonome will recognize the word *non*, and exclude such matches.

Data can be read from CSV files, and the software is flexible enough to accept a range of possible structures. Output data are also written to CSV files. Data that are to be re-used within Taxonome can be saved in a simple format based on JSON (Crockford 2006), which can store structured data, such as nested lists, more conveniently than tabular CSV files. Custom code can be written to convert taxonomic data from other formats. For example, the authors have successfully used data from the Kew grass synonymy database (Clayton et al. 2002), and from the ILDIS legume database (International Legume Database & Information Service 2005). The scripts to read these data sources are available from Taxonome's website.

Taxonome can also retrieve information from a number of web services. For instance, sets of taxa with synonym information can be fetched from the USDA GRIN database (USDA Agricultural Research Service 2012), and names can be matched using the Taxonomic Name Resolution Service (iPlant Collaborative 2012).

## Distributions

Species' distributions are often described by a list of regions where the species occurs, but different data sources may use different sets of regions. The International Taxonomic Database Working Group (TDWG) has defined a set of regions at four different scales, largely following political boundaries, for which GIS data are available (Brummitt et al. 2001). Taxonome includes an index of these regions, with some extra names and groups. This can convert distributions listing names of countries or major regions to sets of TDWG regions, which are more convenient for display or comparison.

The distribution functions are currently only available in the library interface; future versions of the GUI may expose these tools.

## Examples of use

Taxonome has been used in mapping the dominant grass species in different ecoregions. The Kew grass synonymy database (Clayton et al. 2002) was translated into a Taxonome dataset using a custom script, which is available from Taxonome's website. Information from other sources, such as height and photosynthetic pathway, was attached to this within Taxonome. Using diverse literature sources, a set of CSV files was compiled listing the dominant grass species in each ecoregion. From these, the names were extracted and temporarily stored in another CSV file, which was passed to Taxonome. For each of these names from the literature, Taxonome found the accepted name according to Kew's database, and recorded properties of that species. Another custom script cross-referenced the names to produce summary information for each ecoregion, such as the percentage of C_4_ species in the grass flora. This usage case is a specific example of a more general case, that of data compilations of species within survey plots (e.g. Vegbank – http://www.vegbank.org) or species within vegetation formations (e.g. ecoregions).

The ILDIS legume database (International Legume Database & Information Service 2005) stores distribution information for thousands of legume species, by country and region names. The authors use Taxonome to find equivalent sets of regions from the level 3 regions defined by TDWG (Brummitt et al. 2001), allowing us to match geographical information to species traits, and to map the spatial distribution of these traits for hundreds of species. With growing interest in compiling large-scale public trait databases (e.g. http://www.try-db.org), such applications are becoming increasingly feasible. The script to parse the ILDIS database is available from Taxonome's website.

## Availability

Taxonome can be downloaded from http://taxonome.bitbucket.org/ (persistent URL http://purl.org/NET/taxonome).

As an application, Taxonome is available as a package to install on Windows, Mac OS or Linux. To use it as a library, Python 3 is required (Python development team 2012). To run the GUI from source, PySide or PyQt4 is also needed (PySide developers 2012; Riverbank Computing Ltd 2012).

Taxonome is released under the permissive MIT license. Interested users are invited to examine the source code and contribute improvements.
